# Engineered Oxalate Decarboxylase Boosts Activity and Stability for Biological Applications

**DOI:** 10.1021/acsomega.4c11434

**Published:** 2025-03-24

**Authors:** Mirco Dindo, Carolina Conter, Gen-Ichiro Uechi, Gioena Pampalone, Luana Ruta, Angel L. Pey, Luigia Rossi, Paola Laurino, Mauro Magnani, Barbara Cellini

**Affiliations:** † Department of Medicine and Surgery, Section of Physiology and Biochemistry, University of Perugia, 06132 Perugia, Italy; ‡ Protein Engineering and Evolution Unit, Okinawa Institute of Science and Technology (OIST), Onna, Okinawa 904-0495, Japan; § Center of Cooperative Research in Biosciences (CIC bioGUNE) Basque Research and Technology Alliance (BRTA), Bizkaia Technology Park, Building 801A, 48160 Derio, Spain; ∥ Department de Quimica Fisica, Unidad de Excelencia en Quimica Aplicada a Biomedicina y Medioambiente e Instituto de Biotecnologia, Universidad de Granada, Granada 18071, Spain; ⊥ Department of Biomolecular Sciences, University of Urbino “Carlo Bo”, Urbino 61029, Italy; # Institute of Protein Research, Osaka University, Suita, Osaka 565-0871, Japan

## Abstract

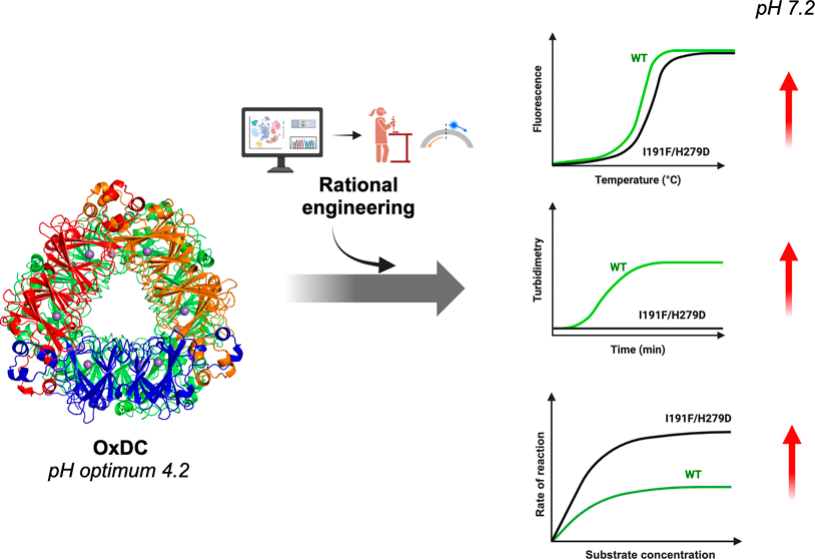

Oxalate decarboxylase (OxDC) from *Bacillus subtilis* is a Mn-dependent hexameric enzyme that converts oxalate to carbon dioxide and formate. Recently, OxDC has attracted the interest of the scientific community due to its biotechnological and medical applications for the treatment of hyperoxaluria, a group of pathologic conditions associated with excessive oxalate urinary excretion caused by either increased endogenous production or increased exogenous absorption. The fact that OxDC displays optimum pH in the acidic range represents a big limitation for most biotechnological applications involving processes occurring at neutral pH, where the activity and stability of the enzyme are remarkably reduced. Here, through bioinformatics-guided protein engineering followed by combinatorial mutagenesis and analyses of activity and thermal stability, we identified a double mutant of OxDC endowed with enhanced catalytic efficiency and stability under physiological conditions. The obtained engineered form of OxDC offers a potential tool for improved intestinal oxalate degradation in hyperoxaluria patients.

## Introduction

Hyperoxaluria is a pathologic condition characterized by increased urinary oxalate excretion.^1−3^ In humans, oxalate is a metabolic end product that can arise from endogenous (glycolate and hydroxyproline metabolism) or exogenous (diet) sources.^3−5^ Interestingly, oxalate homeostasis is also affected by the gut microbiota, which can degrade oxalate and is able to modulate intestinal absorption and excretion.^6^ Hyperoxaluria is the main risk factor for the formation of calcium oxalate stones in the urinary tract, which can progress to nephrocalcinosis and kidney failure.^1,7^ This condition can result from (i) an increased endogenous oxalate production due to genetic alterations in genes involved in glyoxylate/oxalate liver metabolism and, in this case, is called primary hyperoxaluria (PH)^2^ or (ii) alterations of the gastrointestinal tract leading to increased exogenous oxalate absorption, a condition named secondary hyperoxaluria (SH).^1^ A specific form of SH, named enteric hyperoxaluria, is often observed in patients affected by disorders of the gastrointestinal tract and can be the result of an increased bioavailability of dietary oxalate possibly associated with an increased permeability to oxalate of the intestinal epithelium.^3,7,8^ One of the therapeutic approaches proposed for the treatment of hyperoxaluria is the oral administration of oxalate decarboxylase (OxDC) (Uniprot ID: O34714), a nonhuman enzyme that can degrade intestinal oxalate.^9−14^ Indeed, OxDC can reduce the absorption of exogenous oxalate, thus counteracting the basic cause of SH.^10^ In addition, a low oxalate concentration in the gut can promote the intestinal excretion of plasmatic oxalate, thus possibly reducing the burden in PH patients.^8^ This explains why OxDC has attracted great interest from the scientific community. The initial use of OxDC focused on industrial applications^15^ (i.e., the prevention of the formation of oxalate salt deposits in industrial processes such as papermaking and beer production). The potential biomedical application of OxDC has been also extended to the diagnostic level for the determination of oxalic acid concentration in food and complex biological samples such as blood and urine.^12,16−18^

**Figure 1:**
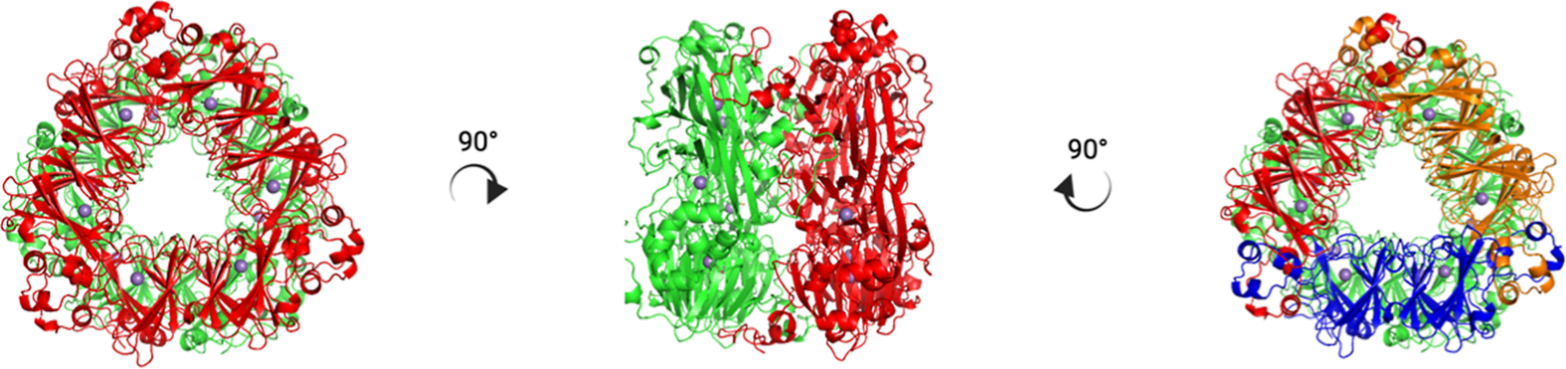
Structural features of *B. subtilis* OxDC. Dimers of each trimeric unit are colored in red and green on the left and central image, while the three monomers forming the trimer are colored blue, orange, and red, on the right image. The images were created by using PyMOL. Molecular Graphics System, Version 1.2r3pre, Schrödinger, LLC,^25^ starting from the crystal of OxDC using the PDB id: 1J58.^19^

OxDC from *Bacillus subtilis* is a homohexamer (a dimer of trimers) (Figure 1) of 264 kDa, which belongs to the cupin superfamily and requires Mn^2+^ and O_2_ to catalyze the conversion of oxalate to formate and CO_2_.^19^ Structurally, each monomer consists of two cupin domains showing a characteristic β-sandwich structure and coordinates two Mn^2+^ ions located in the center of each cupin domain, where the metal interacts with highly conserved amino acids.^19−21^ Recently it has been experimentally proved that two residues, Trp96 and Trp274, are important for catalysis in OxDC. This experimental finding, coupled with theoretical predictions, strongly supports the hypothesis that electron hopping between the C- and N-terminal Mn ions plays a central role in the catalytic mechanism.^22^ Other studies have identified the presence of a channel for oxalate diffusion in the N-terminal domain of the monomers, which can exist in an “open” or “closed” conformation.^21,23^ The major player involved in the structural rearrangement of the channel is a pentapeptide loop formed by residues 161–165, generating a lid important for reaction specificity that contains the proton donor residue (Glu162) and isolates the active site from the solvent during catalysis.^20,24^

Although OxDC shows optimal catalytic activity at acidic pH, its biochemical characterization under physiological conditions has revealed that the enzyme shows detectable activity at neutral pH but also an increased tendency to unfold and aggregate.^26^ These features limit the therapeutic applications of OxDC under conditions of pH and ionic strength typical of a physiological environment.

In this work, we have produced an engineered form of the enzyme more stable and active under physiological conditions as compared with the wild-type counterpart. Specifically, guided by bioinformatic analyses, we have generated and purified a double mutant (I191F/H279D) showing enhanced in vitro thermal stability (*T*_m_ value 5 °C higher that the wild-type form), reduced propensity to aggregation under physiological conditions, and, more importantly, an increased catalytic activity at pH 7.2 (4-fold increase of the *k*_cat_/*K*_M_ value). By modeling approaches, we have also proposed the structural reasons explaining the increased stability of the mutant. Overall, these data indicate that engineered OxDC is more efficient in degrading oxalate under physiological conditions, thus enhancing its potential as biological drug for genetic and nongenetic forms of hyperoxaluria.

## Materials and Methods

### Materials

Potassium oxalate, sodium formate, potassium permanganate, isopropyl-β-d-thiogalactopyranoside, manganese chloride, and imidazole were purchased from Merck Life Science srl (St. Louis, MO, USA). Oligonucleotides for site-directed mutagenesis were purchased from Bio-Fab Research (Rome, Italy). All other chemicals used were of the highest analytical grade.

### Consensus-Based Approach

The consensus-based sequence was calculated using the ConSurf web server (http://consurf.tau.ac.il/2016/).^27^ The homologue sequences of OxDC were collected from the UNIREF90 database and selected by using HMMER (*E*-value cutoff was 0.0001, and the number of iterations was 1). The multiple sequence alignment of 200 homologues sequences (identity from 95% to 35%) was performed by using the MUSCLE (multiple sequence comparison by log-expectation) (Figure S1).^28^ The consensus sequence-based alignment was visualized and analyzed using Jalview 2.10.5 (https://www.jalview.org).^29^

### Molecular Modeling and Electrostatic Potential Maps

Visual inspection of the OxDC crystal structure (PDB id: 1J58) was performed using the software Python-enhanced Molecular Graphics tool (PyMol) (Schrödinger, LLC)^25^ and UCSF Chimera X-1.7 (National Institute of Health, NIH).^30^ The dimeric structure was obtained using the proteins, interfaces, structures and assemblies (PISA) PDBePISA web server,^31^ starting from the available coordinate file of the monomer (PDB id:1J58). The mutants I191F and H279D were prepared using UCSF Chimera X-1.7^30^ with the rotamers tool, through the Dunbrack rotamer library.^32^ The electrostatic maps of the OxDC wild type and mutant H279D were calculated by using the web server APBS-PDB2PQR software suite (https://server.poissonboltzmann.org).^33^ In detail, the analysis of the protonation states of the residues at pH 7.0 was carried out using the PDB2PQR suite tool (using CHARMM as forcefield), which creates an input file for the calculation of the electrostatic potential map using the APBS suite. The graphic visualization of the electrostatic potential maps of the OxDC wild type and H279D mutant was obtained by UCSF Chimera X-1.7.^30^

### Site-Directed Mutagenesis

The selected mutations were introduced by site-directed mutagenesis using the QuikChange II site-directed mutagenesis kit (Stratagene San Diego, California) on the pET24a vector containing the sequence encoding for *B. subtilis* OxDC (Uniprot ID: O34714 endowed with a C-terminal histidine tag (pET24a-OxDC). The oligonucleotides used for the mutagenesis are given in Table S1. All the mutations were confirmed by the entire DNA sequencing.

### Expression and Purification of OxDC and Mutants

His-tagged OxDC wild type and the selected mutants were expressed in *Escherichia coli* and purified by affinity chromatography with minor modifications from the previously published protocol.^26^In detail, *E. coli* BL21 (DE3) cells transformed with the constructs pET24a-OxDC were grown in Luria Broth in a total volume of 0.5 L at 37 °C. Cells were grown with vigorous shaking to an OD of 0.3–0.4 at 600 nm, at which point 5 mM MnCl_2_ was added to the culture to ensure sufficient manganese incorporation, an aspect critical for proper folding and activity of the enzyme.^22,34^ Protein expression was then induced by 0.2 mM IPTG for 16 h at 30 °C. Cells were then harvested by centrifugation and resuspended in lysis buffer (50 mM Tris–HCl pH 8 containing 0.5 M NaCl, 20 mM imidazole, and EDTA-free protease inhibitor cocktail). After sonication, the cell debris was removed by centrifugation (16,000*g* for 30 min at 4 °C). The lysate was loaded on a homemade nickel-affinity resin column (2 mL of resin) equilibrated with 50 mM Tris–HCl at pH 8 containing 500 mM NaCl and 50 mM imidazole. At this point, 10 mL of the same buffer containing 500 mM imidazole was applied. Upon forced dialysis by Amicon ultra 10 devices (10 kDa cutoff) and wash with storage buffer (50 mM Tris–HCl pH 8, 500 mM NaCl and 5 mM DTT), OxDC wild type and the mutants were conserved at −20 °C. The purification level was evaluated by sodium dodecyl sulfate-polyacrylamide gel electrophoresis (SDS-PAGE), using 1 μg of OxDC per lane on 10% acrylamide gel (Figure S4A, B). Protein concentration was measured by the absorbance at 280 nm using the extinction coefficient of 42,340 M^–1^ cm^–1^, and the purification yield of each species is reported in Figure S4C.

### Spectroscopic Measurements

Absorption measurements for protein quantification were carried out using a JASCO V-750 spectrophotometer on 1 cm path length quartz cuvettes^35^ (JASCO Europe S.r.l.). 8-Anilino-1-naphthalene sulfonate (ANS) fluorescence emission spectra were recorded on a JASCO FP8200 spectrofluorometer equipped with a thermostatically controlled cell holder by using 1 cm path length quartz cuvettes. Excitation was set at 365 nm with both the excitation and emission slits set at 5 nm. The ANS-binding experiments were performed at 25 °C using a 1 μM protein concentration in 16 mM Tris–HCl, 140 mM NaCl, pH 8.0. The changes in turbidity were monitored by measuring the absorbance at 500 nm as a function of time using a MultiSkan SkyHigh microplate reader (Thermo Fisher Scientific) at 37 °C in PBS buffer at a 1–3 μM protein concentration in a final volume of 200 μL.

### Enzymatic Activity Measurements

OxDC wild type and mutants’ activity at different pH was determined by measuring the product formate using potassium permanganate. Potassium permanganate is a chemiluminescent reagent often used to detect organic molecules.^17^ It displays absorption maxima at three different wavelengths, specifically at 525, 545, and 569 nm, and it was reported^36^ that the addition of formate to 1 mM potassium permanganate solution leads to a significant decrease of absorbance.

The measurements were initially setup in the presence of different formate concentrations and at a fixed potassium permanganate concentration of 1 mM by checking the change in the absorbance signal at the three permanganate maxima (i.e., 525, 545, and 569 nm). Based on the results of these experiments, we chose to measure the time-dependent changes of the signal at 545 nm to detect formate produced by the enzymatic reaction of OxDC. We then checked if the substrate (potassium oxalate) and/or the reaction buffer (acetate buffer 52 mM + 140 mM NaCl) could interfere with the assay. As reported in Figure S5A,B, neither oxalate nor acetate interferes with the absorbance change of potassium permanganate resuspended in KP 100 mM pH 8.0 (Figure S5A). Nevertheless, we noticed that a slight change in the absorbance signal at 545 nm is observed in blank assay mixtures in the absence of oxalate. For that reason, the specific decrease was measured for each assay mixture and subtracted from the measurement of each sample. In detail, the kinetic parameters were determined at two different pH values. At pH 4.2, the reactions were performed in 52 mM sodium acetate in the presence of NaCl 140 mM, using 0.1 μM enzyme at 37 °C for 5–10 min in the presence of different potassium oxalate concentrations (0–150 mM). At pH 7.2, the reactions were performed in PBS 1× using 0.3–0.5 μM enzyme at 37 °C for 25–35 min in the presence of different concentrations (0–150 mM) of potassium oxalate and in the presence of Mn_2_Cl. In both cases, 100 μL of the reaction mix was stopped by increasing the pH with the addition of 100 μL of premixed KP 100 mM pH 8.0 containing 2 mM potassium permanganate (final volume of 200 μL), and the absorbance at 545 nm for the first 0–200 s was measured by using the MultiSkan SkyHigh microplate reader at 25 °C. The permanganate along with the neutral pH inactivates the enzyme. The kinetic parameter experiments on the OxDC WT and on the double mutant I191F/H279D at pH 7.2 have also been performed in the presence of exogenous Mn(II) (2 mM).

To normalize for any possible difference due to the buffer used in the enzymatic reaction, we calculated a specific calibration curve for each experimental condition (GraphPad Software, Boston, Massachusetts USA, www.graphpad.com).

### Thermal Shift Assay

The thermal shift assay was used to estimate and compare the thermal stability of the OxDC wild type and mutants using a high-throughput differential scanning fluorimetry assay. We used SYPRO orange 5000× (Sigma-Aldrich) as the fluorescent dye. The fluorescence of the dye is quenched in aqueous solution but increases when it binds to hydrophobic regions exposed upon protein unfolding. We used a final concentration of 5x SYPRO orange dye (1:1000 v/v) with 1 μM protein in a total volume of 20 μL. The experiments were performed by using a StepOnePlus real-time instrument (Thermo Fisher Scientific) in 96-well plates. The thermal shift assay experiments on the OxDC WT and on the double mutant I191F/H279D have also been performed in the presence of exogenous Mn(II) (2 mM).

Data were analyzed using a script and repeated at least 3 times using the same experimental conditions.

### Proteolysis Experiments

0.5 mg of OxDC WT and I191F/H279D were resuspended in PBS 1× pH 7.2 in the presence of 10 μg of proteases pancreatin (Sigma-Aldrich, P3292) or α-chymotrypsin (Sigma-Aldrich, C4129) (ratio OxDC/protease 50:1**)**. The reaction mix (2 mL) was incubated for 5 h at 37 °C. 100 μL of reaction mix was withdrawn at different time points (0, 60, 120, 150, and 300 min), and the residual decarboxylase activity was tested by using the potassium permanganate assay. The oxalate concentration used to test OxDC residual enzymatic activity in the presence of proteases was 100 mM. The *t*_1/2_ and the *k*_d_ values were calculated using a single exponential decay equation using GraphPad Prism 10.2.1 (GraphPad Software, Boston, Massachusetts USA, www.graphpad.com).

### Determination of the Kinetic Stability of the OxDC Wild Type and Mutant I191F/H279D

To determine the kinetic stability of OxDC wild type and the mutant I191F/H279D, we have incubated the enzymes at 37 °C in PBS pH 7.2 at a 1 μM protein concentration and measured the residual activity at different incubation times (between 0 and 1500 min). For all measurements, the value of the activity of the proteins at time 0 was set as 100%. The *t*_1/2_ and the *k* values were calculated by fitting the data to a single exponential decay equation using GraphPad Prism 10.2.1 by setting the plateau value fixed at 0 (GraphPad Software, Boston, Massachusetts USA, www.graphpad.com).

### Statistical Analysis

Data were analyzed using GraphPad Prism 10.2.1 (GraphPad Software, Boston, Massachusetts USA, www.graphpad.com). All of the data presented in this work represent the mean ± SD of at least two independent experiments.

## Results

### Rational Engineering of OxDC Using the “Consensus-Based Approach”

Recent studies on the molecular features of *B. subtilis* OxDC reported that at neutral pH, the protein is relatively unstable and retains poor residual activity. A well-accepted and effective strategy to improve protein stability and activity is the “consensus-based” approach.^37^ Consensus mutations are reversions of some protein residues to the ancestral amino acids, an approach successfully applied to improve the overall stability and/or increase the catalytic efficiency of several proteins.^37−39^ We started by generating the consensus sequence of *B. subtilis* OxDC.

**Figure 2:**
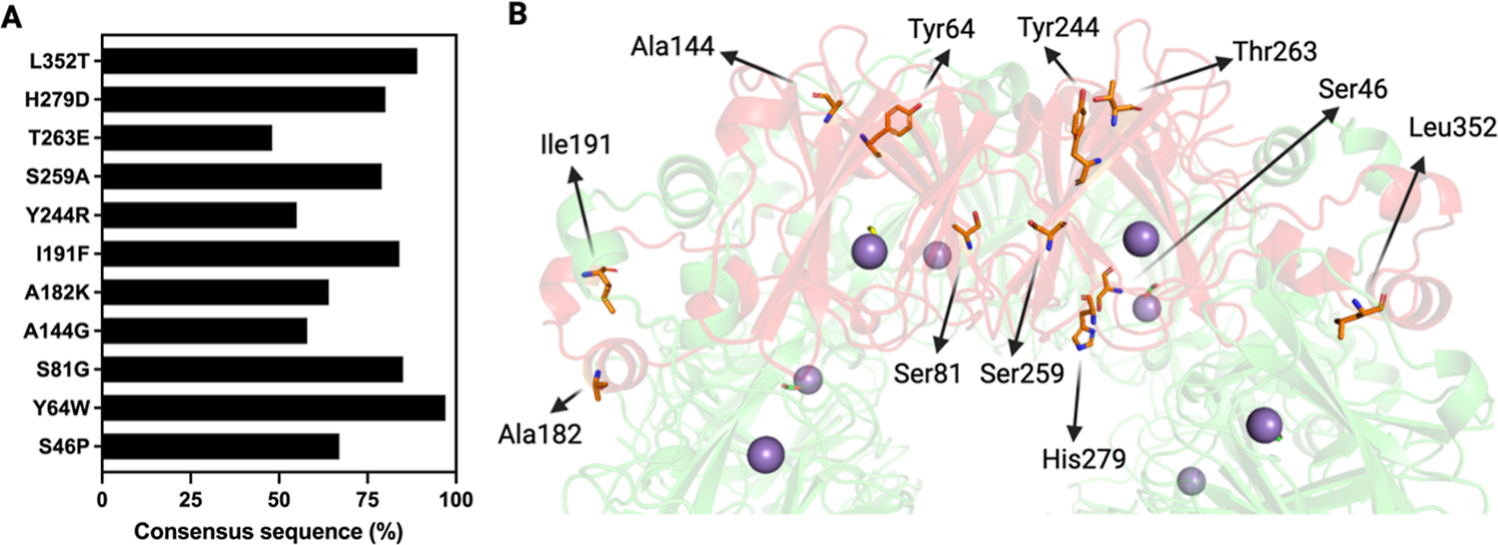
Consensus-based analysis of *B. subtilis* OxDC. (A) Output of the consensus-based analysis obtained by using the ConSurf web server.^27^ The bar graph shows the “consensus” percentage of the selected amino acid substitutions on the OxDC sequence orthologues selected in this study. (B) Position of the amino acid residues selected as targets for mutagenesis within the OxDC structure (PDB id: 1J58).^19^ The monomer used to show the position of the consensus residues selected is highlighted and colored red, and each target residue is shown as orange sticks, while the remaining monomers are all colored green. The image was created by using PyMOL Molecular Graphics System, Version 1.2r3pre, Schrödinger, LLC.^40^

We selected a set of 11 mutations involving residues belonging to different protein domains (Figure 2A) based on the percentage of the consensus analysis (i.e., residues with the highest frequency at individual positions in the multiple sequence alignment reported in Figure S1). From a structural point of view, mutation sites are spread over the entire dimer, being localized on the monomer surface, in the monomer core, or in the proximity of the monomer–monomer interface of each trimer (Figure 2B).

### Biochemical Properties of Single Mutants

Each of the 11 selected mutations was introduced on the OxDC sequence by site-directed mutagenesis on the pET24a^(+)^-OxDC vector for bacterial expression.^26^ Seven protein variants were purified in high amounts from the soluble fraction and gave yields comparable to those of the wild type or even higher (Figure S3). On the other hand, the variants Y64W, A144G, A182K, and L352T were mainly present in the insoluble fraction of the cellular lysate, suggesting that these mutations might affect the correct folding of the protein. For this reason, they were excluded from subsequent analyses.

**Figure 3:**
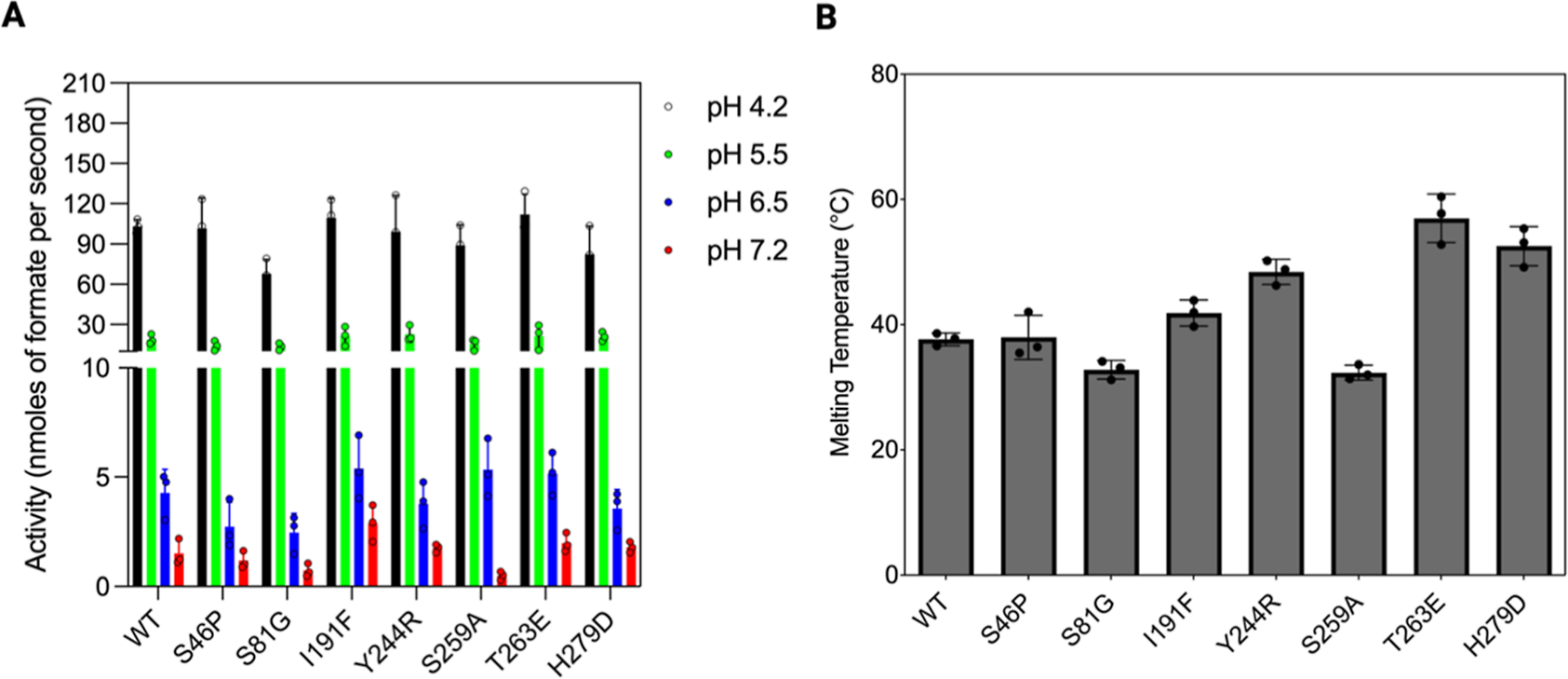
Functional and structural features of the selected single mutants. (A) Activity (expressed as nmoles of formate per second) of the OxDC wild type and of the single mutants, measured at different pH values (as indicated in the figure legend) at 37 °C in 52 mM sodium acetate pH 4.2, sodium acetate pH 5.5, 26 mM Bis-Tris pH 6.2, and PBS 1× pH 7.2 using 50 mM potassium oxalate. (B) Bar charts of the values of thermal stability of the OxDC wild type and of the single mutants obtained by monitoring the changes of the CD signal at 222 nm in 16 mM Tris–HCl, 140 mM NaCl at pH 7.2.

The specific activities at different pH values and melting temperatures of the single mutants are shown in Figure 3. We found that the mutants I191F, Y244R, T263E, and H279D display decarboxylase activity at pH 6.5 and 7.2 higher than the OxDC wild type (Figure 3A). On the other hand, the mutations S46P, S81G, and S259A do not increase the activity of OxDC at physiological pH. Analyzing the structural localization of the mutated residues, we noticed that beneficial mutations are mainly located on the protein surface (Y244R, T263E, and H279D) or at the monomer–monomer interface (I191F). Notably, mutations involving residues located in the core of the OxDC monomer (such as S46, S81, and S259) do not increase activity at neutral pH and do not affect or even decrease thermal stability, thus indicating that alterations of the corresponding regions could possibly interfere with the proper folding of the monomeric subunits.

### Generation and Characterization of Second-Generation Mutants by Combinatory Mutagenesis

**Figure 4:**
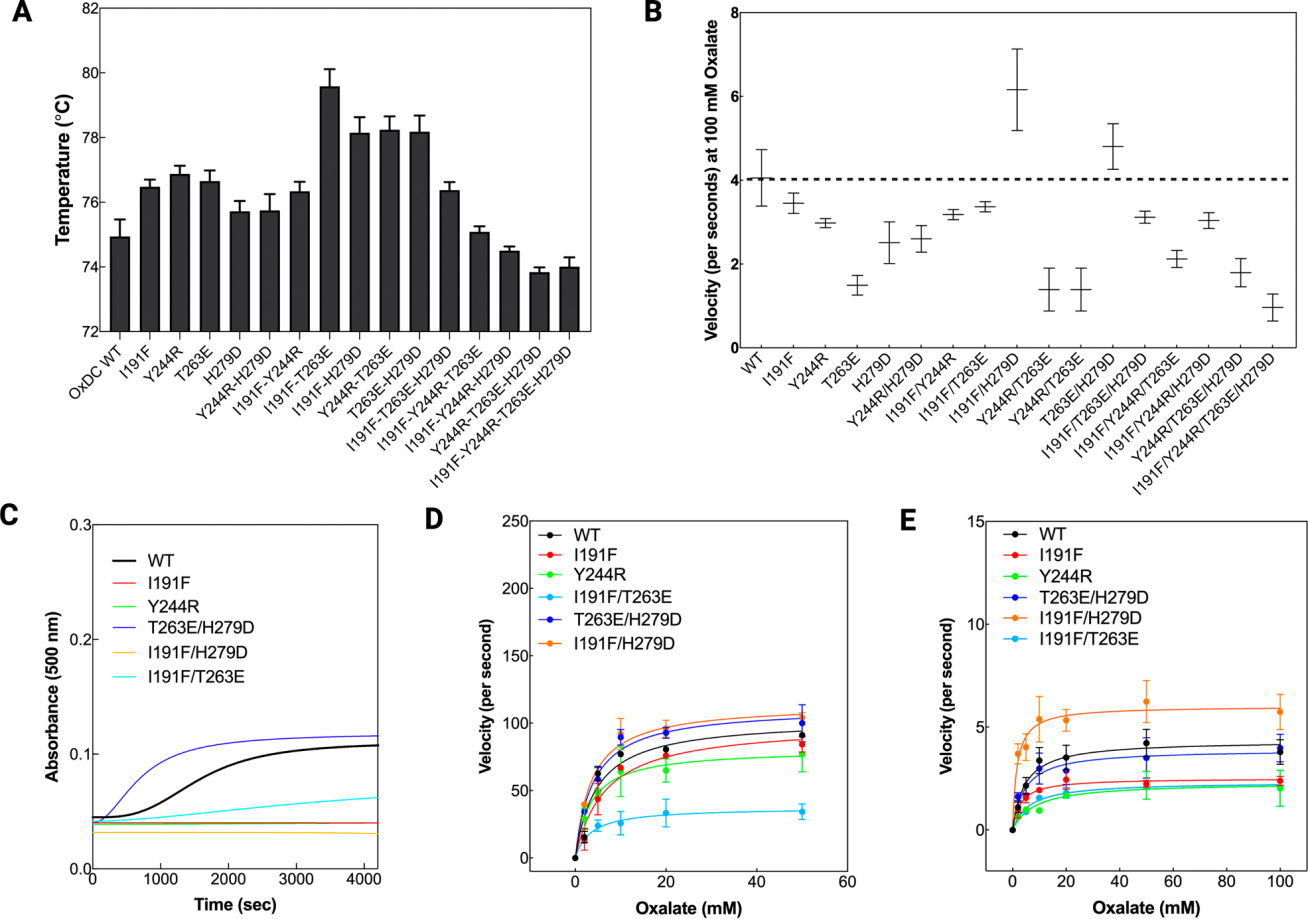
Structural and functional features of the selected OxDC mutants. (A) Experimental thermal shift values (°C) obtained by using the SYPRO orange probe. The experiments were performed by using 1 μM protein and 5× SYPRO orange as the final concentration in PBS 1× pH 7.2. Each experiment was repeated at least in triplicate for each mutant. (B) Turnover number expressed as velocity (seconds) using PBS 1× at pH 7.2 using 100 mM oxalate as the substrate. The dotted line indicates the OxDC wild type value of activity, used as reference. (C) Turbidimetry experiments on the selected mutants performed by monitoring time-dependent changes in the absorbance at 500 nm. The experiments were performed by using a 2–3 μM protein concentration at 37 °C in PBS 1×. (D) Kinetic parameters of OxDC wild type and mutants measured by using a 0.1 μM protein concentration in sodium acetate 52 mM, NaCl 140 mM pH 4.2 at 37 °C. The curves represent the fitting to the Michaelis–Menten equation. (E) Kinetic parameters of the OxDC wild type and mutants measured by using a 0.3 μM protein concentration in PBS 1× pH 7.2 at 37 °C. The curves represent the fitting to the Michaelis–Menten equation. Data were collected and analyzed in triplicate.

We combined the four single beneficial mutations previously identified (I191F, Y244R, T263E, and H279D) to produce the whole set of combinations of double and triple mutants as well as the quadruple mutant. We first evaluated the effects of combinatory mutagenesis on the thermal stability and catalytic activity at a saturating substrate concentration. Interestingly, as shown from the data in Figure 4A, the best results were obtained from double mutants, which show an increased melting temperature as compared to single, triple, and the quadruple mutant. Specifically, the mutants I191F/T263E, I191F/H279D, Y244R/T263E, and T263E/H279D show an increase in thermostability of 3–6 °C as compared to the OxDC wild type.

Based on the overall data obtained and reported in panels A and B of Figure 4, we decided to further characterize the species showing an increase in melting temperature associated with an increased or almost preserved activity at pH 7.2: I191F, Y244R, T263E/H279D, I191F/H279D, and I191F/T263E.

Since the two major concerns about the OxDC wild type under physiological conditions are related to its increased aggregation tendency and decreased catalytic efficiency, we focused on the stability and kinetic features of the selected mutants. Turbidimetry experiments revealed that OxDC wild type shows a considerable aggregation propensity at 37 °C under conditions mimicking physiological pH and ionic strength (PBS pH 7.2) with a *t*_1/2_ = 1567 ± 21 s (Figure 4C). Interestingly, all the selected mutants showed slight or no aggregation propensity under physiological conditions as compared to the OxDC wild type, except the double mutant T263E/H279D that displays an increased aggregation rate (*t*_1/2_ = 699 ± 44 s).

By calculating the kinetic parameters of the mutants at pH 4.2, which represents the optimum of the enzyme (Figure 4D), we confirmed that all species retain detectable activity with values of catalytic efficiency for the decarboxylase reaction similar to or lower than that of the wild type (Figure 4D and Table 1). However, a different scenario appeared upon the determination of the kinetic parameters under physiological conditions. At pH 7.2, most of the single and double mutants display a decreased catalytic efficiency as compared to the OxDC wild type, driven by altered *k*_cat_ and *K*_M_ values, while the mutant I191F/H279D shows a catalytic efficiency increased by 4-fold as compared to the wild-type counterpart (Table 1). To confirm the data and to exclude any metal leaking for the OxDC wild type and mutant I191F/H279D, we calculated the kinetic parameters in the presence and absence of 2 mM Mn_2_Cl at pH 7.2. As reported in Figure S2, we did not observe any difference in the kinetic parameter values with and without exogenous Mn(II).

**Table 1:** Kinetic Parameters of the OxDC Wild Type and Mutated Proteins Obtained Using Sodium Acetate 52 mM, NaCl 140 mM pH 4.2 or PBS 1× pH 7.2 at 37 °C

	protein	*k*_cat_ (s^–1^)	*K*_M_ (mM)	*k*_cat_/*K*_M_ (s^–1^ mM^–1^)
pH 4.2				
	wild type	103 ± 15	7 ± 2	15 ± 8
	I191F	101 ± 12	7 ± 2	14 ± 7
	Y244R	95 ± 10	5 ± 1	19 ± 4
	H279D	58 ± 7	6 ± 2	10 ± 3
	I191F/T263E	37 ± 5	5 ± 1	7 ± 2
	I191F/H279D	110 ± 11	5 ± 2	22 ± 10
	T263E/H279D	111 ± 10	5 ± 1	22 ± 8
pH 7.2				
	wild type	3.9 ± 0.5	4.4 ± 1.0	0.89 ± 0.22
	I191F	3.4 ± 0.3	2.3 ± 0.9	1.47 ± 0.55
	Y244R	2.5 ± 0.5	6.5 ± 2.4	0.38 ± 0.15
	H279D	2.4 ± 0.4	5.3 ± 2.2	0.45 ± 0.20
	I191F/T263E	1.8 ± 0.2	5.2 ± 2.0	0.34 ± 0.13
	I191F/H279D	6.0 ± 0.9	1.6 ± 0.4	3.75 ± 1.09
	T263E/H279D	4.5 ± 0.5	3.8 ± 1.1	1.18 ± 0.25

**Figure 5:**
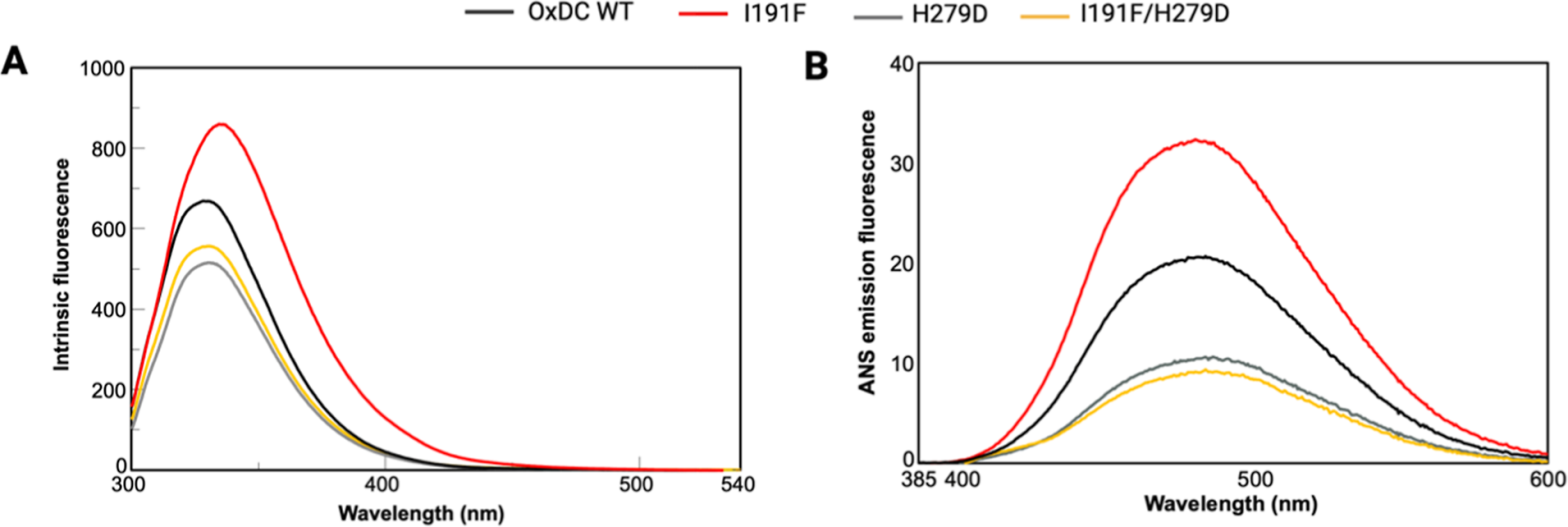
Spectral features of the double mutant I191F/H279D and the single mutants I191F and H279D compared to the OxDC wild type. (A) Intrinsic emission fluorescence spectra of the selected mutants and OxDC wild type registered in PBS 1× using a 0.25 μM protein concentration at 25 °C. The λ_exc_ was 280 nm, and the emission fluorescence was recorded from 300 to 540 nm. (B) ANS emission fluorescence spectra of the selected mutants and OxDC wild type registered in PBS 1× using a 1 μM protein concentration at 25 °C. The λ_exc_ was 365 nm, and the emission fluorescence was recorded from 380 to 600 nm.

Based on the data obtained, we pinpointed the I191F/H279D mutant as the best candidate and decided to characterize the structural features of this mutant form in more detail. We included in the structural analysis the two single mutants I191F and H279D to dissect the contribution of each amino acid change to the structural properties of the double mutant. The comparison of intrinsic fluorescence spectra (which report on the microenvironment of the aromatic side chains) as well as of the ANS fluorescence spectra (which report on the presence of hydrophobic surfaces) provided evidence that the I191F/H279D mutant displays some conformational changes as compared with the OxDC wild type. As shown in Figure 5A,B, the main alterations are related to (i) a consistent decrease (∼1.4-fold) of the maximum intensity in the intrinsic fluorescence spectra and (ii) a ∼ 2-fold decrease in ANS emission fluorescence intensity along with a 4 nm red shift of the emission maximum.

In addition, by analyzing the spectroscopic data of the single mutants, we noticed that the double mutant I191F/H279D shows spectroscopic features similar to the single mutant H279D, whereas the mutation of Ile191 alone seems to induce more remarkable conformational changes to the OxDC structure. On the other hand, the kinetic parameters of the single mutants at pH 7.2 indicate that each of the I191F and H279D single mutations (Table 1) does not increase the *k*_cat_ value or decrease the *K*_M_ value of the enzyme, thus supporting the view that the increased catalytic efficiency of the double mutant is due to subtle changes that occur at the active site as a consequence of a combined effect of the two mutations.

The finding that both I191F and H279D mutations improve OxDC thermal stability (as previously discussed and reported in Figure 4A), although to a different extent, would suggest that the increased activity of the double mutant could be a secondary effect of the overall increased thermal stability and/or decreased aggregation propensity under physiological conditions, possibly as a consequence of a different and more stable conformation of the mutated protein.

To test this hypothesis and gain more insight into the stabilizing effects of the two mutations, we compared OxDC wild type and the double mutant for their sensitivity to proteolytic cleavage and thermal stress.

The results shown in Figure S3 indicate that the double mutant I191F/H279D displays a slightly increased stability against the proteolytic cleavage mediated by the proteases chymotrypsin and pancreatin (Figure S3A,B) and an increased kinetic stability under thermal stress (Figure S3C). By fitting the residual activity of the proteins incubated at 37 °C at different times, we found that the decay constant (*k*) of the double mutant is 35% lower compared to that of the wild-type counterpart. These results suggest that the double mutant could be endowed with a slightly increased stability in a biological environment as compared to the wild-type counterpart due to a structural change caused by the combination of the two mutations.

## Discussion

OxDC is an enzyme endowed with high potential for industrial applications, in particular for the treatment of hyperoxaluria, a group of pathologic conditions associated with increased oxalate excretion by either genetic or environmental causes, resulting in the formation of oxalate stones mainly in the kidneys.^1,3,9^ The oral administration of OxDC is one of the therapeutic approaches proposed for the treatment of hyperoxaluria to degrade intestinal oxalate, thus preventing intestinal absorption from exogenous sources or favoring intestinal excretion of the endogenous pool. The main limitations related to the use of OxDC as a therapeutic are the very low decarboxylase activity at neutral pH,^26^ along with an enhanced propensity to unfolding and aggregation,^26^ which compromise the efficiency of intestinal oxalate degradation.^41^

**Figure 6:**
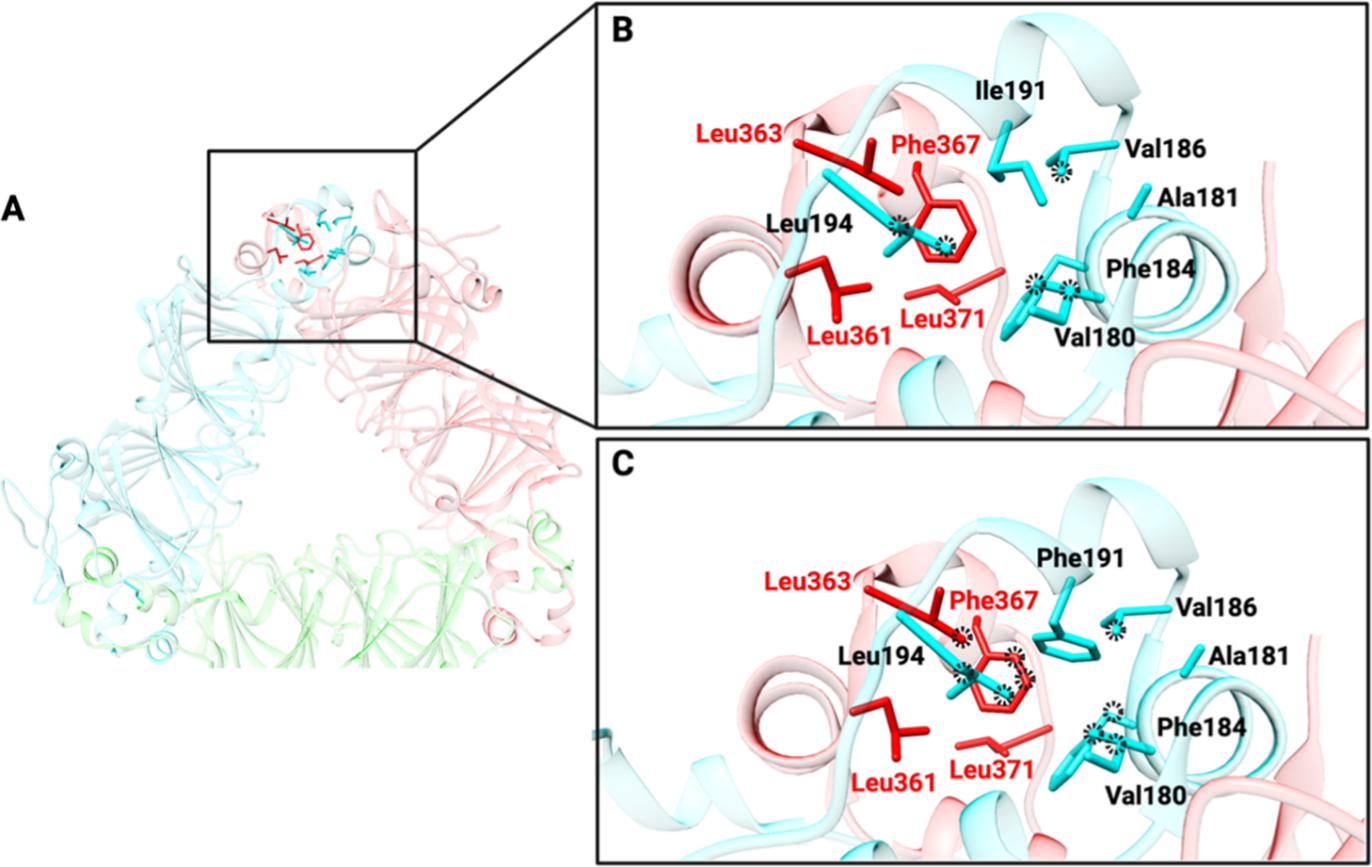
*In silico* analysis of the effects of the I191F mutation. (A) Localization of the residue Ile191 in the OxDC crystal structure.^19^ The OxDC dimers are colored in red, cyan, and green. (B) Zoom-in of the interactions mediated by Ile191 (cyan) with the surrounding residues. The interactions are highlighted using black dot circles. (C) Molecular modeling of the mutation Ile191Phe and analysis of the interactions mediated by Phe191 with the surrounding residues. The mutant I191F was prepared by using the tool “rotamers”^32^ present in the program UCSF Chimera X-1.7.^30^

Here, we performed the engineering of OxDC from *B. subtilis* through a consensus-guided strategy. Upon the first screening of the effects of single selected mutations on the specific activity of the enzyme, we chose the favorable ones and started a combinatorial mutagenesis study to foster protein stability by exploiting possible nonadditive effects of multiple mutations.^42,43^ We obtained the I191F/H279D double mutant, which shows an increased catalytic efficiency at neutral pH, increased thermostability, and almost undetectable propensity to aggregation under physiological conditions of ionic strength and pH. These effects are similar to those obtained on other enzymes using analogous strategies.^11,37,44−47^ A comparative analysis of the effects of each single mutation indicates that I191F and H279D increase the OxDC melting temperature. The crystal structure of the protein shows that Ile191 and His279 are located far from the active site. Ile191 belongs to a region of the cupin domain I that forms a claw-like protrusion directly involved in intersubunits contacts (Figure 6A). The hydrophobic cluster comprising Ile191, Ile180, Ala181, Val186, and Ile194 along with Leu361, Leu363, Phe367, and Leu371 of the neighboring subunit stabilizes a region critical for the stabilization of the OxDC quaternary structure. In fact, the trimeric layers of the hexamer are stabilized by the interlocking claw-like α-helical protrusions of adjacent monomers.^19,48^ Interestingly, the Ile191 side chain mainly interacts with residues located on the same subunits, as reported in Figure 6B. However, the substitution of Ile in position 191 with a bulky Phe residue is predicted to increase the total number of interactions, especially those between the two subunits of the trimer (Figure 6C) without generating steric hindrance with the surrounding environment. In detail, Phe191 directly interacts with Leu363 and Phe367, two residues located on the adjacent subunits, playing an important role in stabilizing the trimeric layers of the OxDC hexamer. This predicted effect could explain the increased thermostability of the single mutant.

**Figure 7:**
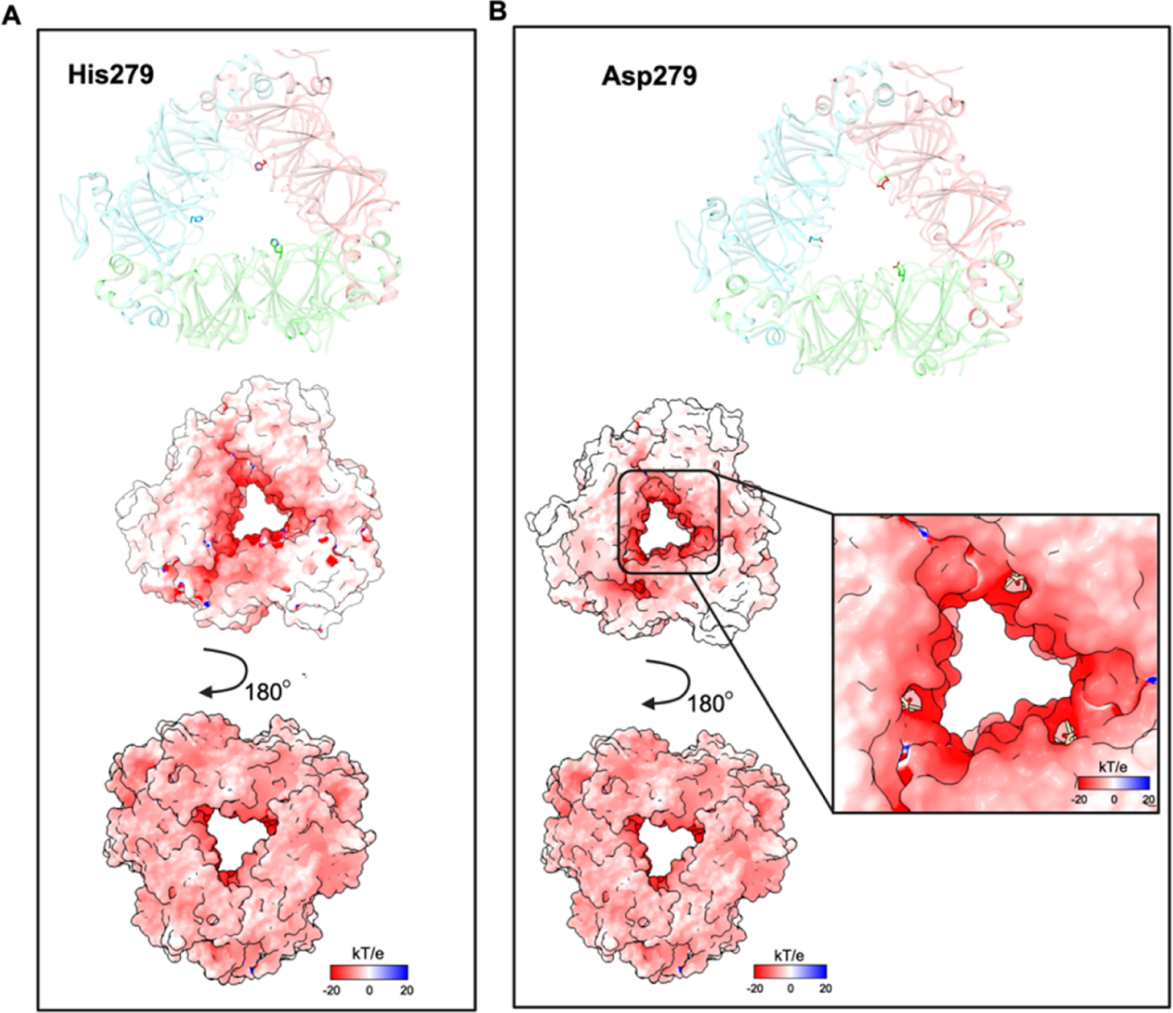
Electrostatic potential maps of OxDC wild type and the H279D mutant. (A) Location of the His279 residues in the crystal structure^19^ and electrostatic potential map of OxDC wild type. (B) Electrostatic potential map of mutant H279D. The mutant was prepared using the tool “rotamers” present in UCSF Chimera X-1.7.^30^ The electrostatic maps were calculated by using the software suite APBS-PDB2PQR at pH 7.2 (https://server.poissonboltzmann.org/pdb2pqr) at 37 °C and visualized using UCSF Chimera X-1.7.^30^ The scale of electrostatics is expressed as *kT*/*e* (−20 and +20).

His279 is part of the cupin domain II and is located on the OxDC surface, where it coordinates solvent molecule and forms a hydrogen bond with Phe315 mainchain.^19^ As reported in Figure 7A, the electrostatic potential map of the OxDC wild type at pH 7.0 shows interesting chemical–physical features: a large negatively charged patch on one side of the surface along with a large uncharged patch on the other side. The same analysis on the H279D mutant (Figure 7B) reveals that the presence of three Asps slightly decreases the area of the uncharged patches along the surface, possibly explaining the low signal of the probe ANS reported in Figure 5B, which indicates a decreased presence of hydrophobic surfaces. In this regard, it is of note that also the double mutant shows a reduced exposure of hydrophobic surfaces with a concomitant increase of negatively charged surfaces and that the H279D mutation is probably the main contributor to this effect. This can provide a possible explanation for the reduced aggregation tendency under physiological conditions shown in Figure 4C.

From the functional point of view, we observed a ∼ 4-fold increase in catalytic efficiency of the I191F/H279D double mutant with respect to the OxDC wild type. Although at present it is not possible to provide a molecular explanation for this result, the increased activity could be a secondary effect due to the stabilization of a catalytic intermediate or transition state during catalysis at neutral pH.^49−51^ It must be mentioned that although in silico analyses provide a structural explanation of the effects observed in vitro, the overall stability depends on several intrinsic features of the proteins. Indeed, the flexibility of its component stretches of amino acids, long-range interactions such as networks of hydrogen bonds, and an increased number of hydrophobic interactions can influence the overall resistance to external stresses, finally resulting in a different half-life in cellular and noncellular systems.^50−52^

In conclusion, we obtained an engineered form of OxDC from *B. subtilis*, showing improved properties in terms of activity and stability under physiological conditions. Our data could help in ameliorating currently available drugs based on OxDC administration, although studies in animal models will be necessary to assess the effects of engineering on the in vivo performance of the enzyme.

## Supporting Information Available

Primers used in this study to generate the OxDC mutants, details about the multiple sequence alignments and the consensus mutations selected, kinetic parameters curves and values obtained in the presence and absence of MnCl_2_ for OxDC wild type and the double mutant I191F/H279D, residual activity of OxDC and the double mutant I191F/H279D in the presence of proteases and under thermal stress, SDS-PAGE and yield obtained of all the proteins involved in the study, and data regarding the setup of the assay for detecting the OxDC activity using potassium permanganate (PDF)
Multiple sequence alignment of OxDC obtained using MUSCLE (TXT)
