# Supplementary material for: Engineered Oxalate Decarboxylase Boosts Activity and Stability for Biological Applications
Source: ACS Omega. 2025 Mar 24;10(12):12375–84. doi: 10.1021/acsomega.4c11434 (PMC11966277; doi:10.1021/acsomega.4c11434)
Supplement: Supplementary file 1 — ao4c11434_si_001.pdf [file ao4c11434_si_001.pdf]

Engineered Oxalate decarboxylase boosts activity and stability for biological applications

Mirco Dindo[1,2*], Carolina Conter[3], Gen-ichiro Uechi[2], Gioena Pampalone[1], Luana Ruta[1], Angel L. Pey[4], Luigia Rossi[5], Paola Laurino[2,6], Mauro Magnani[5] and Barbara Cellini[1*]

[1]Department of Medicine and Surgery, Section of Physiology and Biochemistry, University of Perugia, 06132, Perugia, Italy
[2]Protein Engineering and Evolution Unit, Okinawa Institute of Science and Technology (OIST), Onna, Okinawa 904-0495, Japan
[3]Center of Cooperative Research in Biosciences (CIC bioGUNE) Basque Research and Technology Alliance (BRTA), Bizkaia Technology Park, Building 801A, 48160 Derio, Spain
[4]Department de Quimica Fisica, Unidad de Excelencia en Quimica Aplicada a Biomedicina y Medioambiente e Instituto de Biotecnologia, Universidad de Granada, Granada, Spain
[5]Department of Biomolecular Sciences, University of Urbino "Carlo Bo", Urbino, Italy
[6]Institute of protein Research, Osaka University, Suita, Osaka 565-0871, Japan
*Correspondence: Mirco Dindo (mirco.dindo@unipg.it), Barbara Cellini (barbara.cellini@unipg.it)

Table S1. Primer sequences used for mutagenesis of OxDC.

	Forward primer sequence	Reverse primer sequence
S46P	5'-taaagctgaatttcatgttgggaacggtaccatggtcggtc-3'	5'-gaccgaccatggtaccgttcccaacatgaaattcagcttta-3'
S81G	5'-acgcatgttaacgcccgccaggttctcgc-3'	5'-gcgagaacctggcgggcgttaacatgcgt-3'
I191F	5'-ccggcaggttgctgaactcctctttggtaac-3'	5'-gttaccaaagaggagttcagcaacctgccgg-3’
Y244R	5'-tggtgctatccgcaatacgcaccttgccaccttcgc-3'	5'-gcgaaggtggcaaggtgcgtattgcggatagcacca-3'
S259A	5'-cggtcaccagcgcggccgcaatggtcttgc-3'	5'-gcaagaccattgcggccgcgctggtgaccg-3'
T263E	5'-cgcacccggttcaacctccaccagcgcgctcgc-3'	5'-gcgagcgcgctggtggaggttgaaccgggtgcg-3'
H279D	5'-gtactgccattcgtcggtgttcgggtgcc-3'	5'-ggcacccgaacaccgacgaatggcagtac-3'

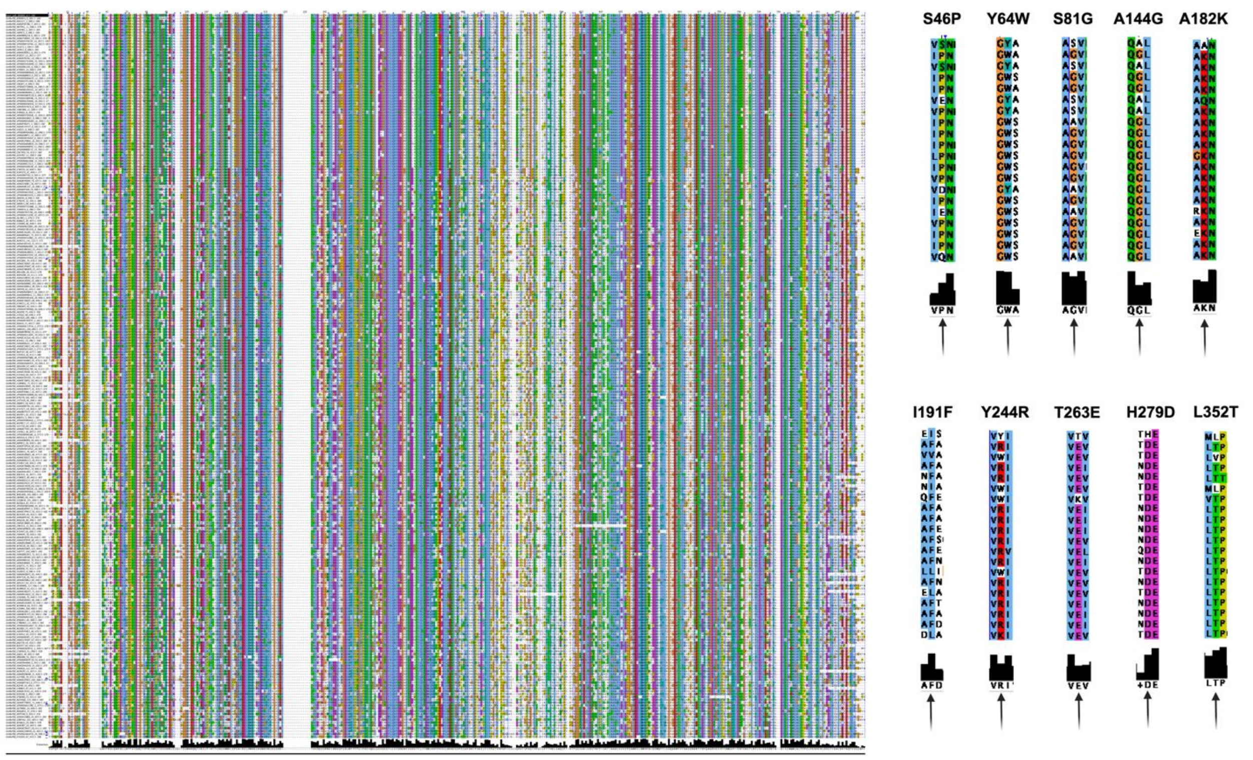

Figure S1. Multiple sequence alignment of OxDC obtained using MUSCLE webserver and visualized using Jalview 2.10.5. The homologues sequences of OxDC were collected from UNIREF90 database and selected by using HMMER. Zoom-in of the selected consensus mutations. If more info about the consensus percentage is needed, please refer to the multiple sequence alignment which is available as supplementary data file or Fig 2 of the main manuscript.

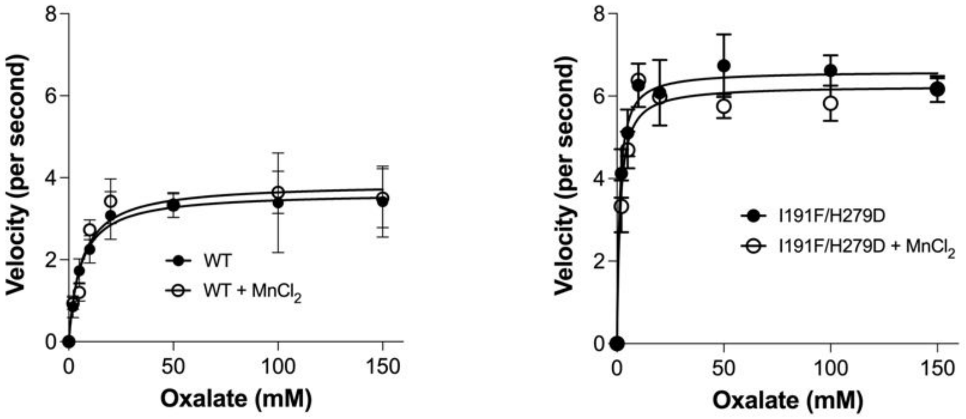

	k_{cat} (s^{-1})	K_M (mM)	k_{cat}/K_M ($s^{-1}mM^{-1}$)
**WT**	3.7 ± 0.4	4.5 ± 0.9	0.82 ± 0.18
**WT + $MnCl_2$**	3.9 ± 0.5	4.3 ± 0.8	0.90 ± 0.17

	k_{cat} (s^{-1})	K_M (mM)	k_{cat}/K_M ($s^{-1}mM^{-1}$)
**I191F/H279D**	6.6 ± 0.8	1.5 ± 0.3	4.4 ± 1.0
**I191F/H279D + $MnCl_2$**	6.2 ± 0.9	1.4 ± 0.3	4.4 ± 1.1

Figure S2. Kinetic parameters of OxDC WT and I191F/H279D obtained in the absence or presence of Mn(II) (as $MnCl_2$) in PBS 1X pH 7.2 using 0.4 μM enzyme concentration.

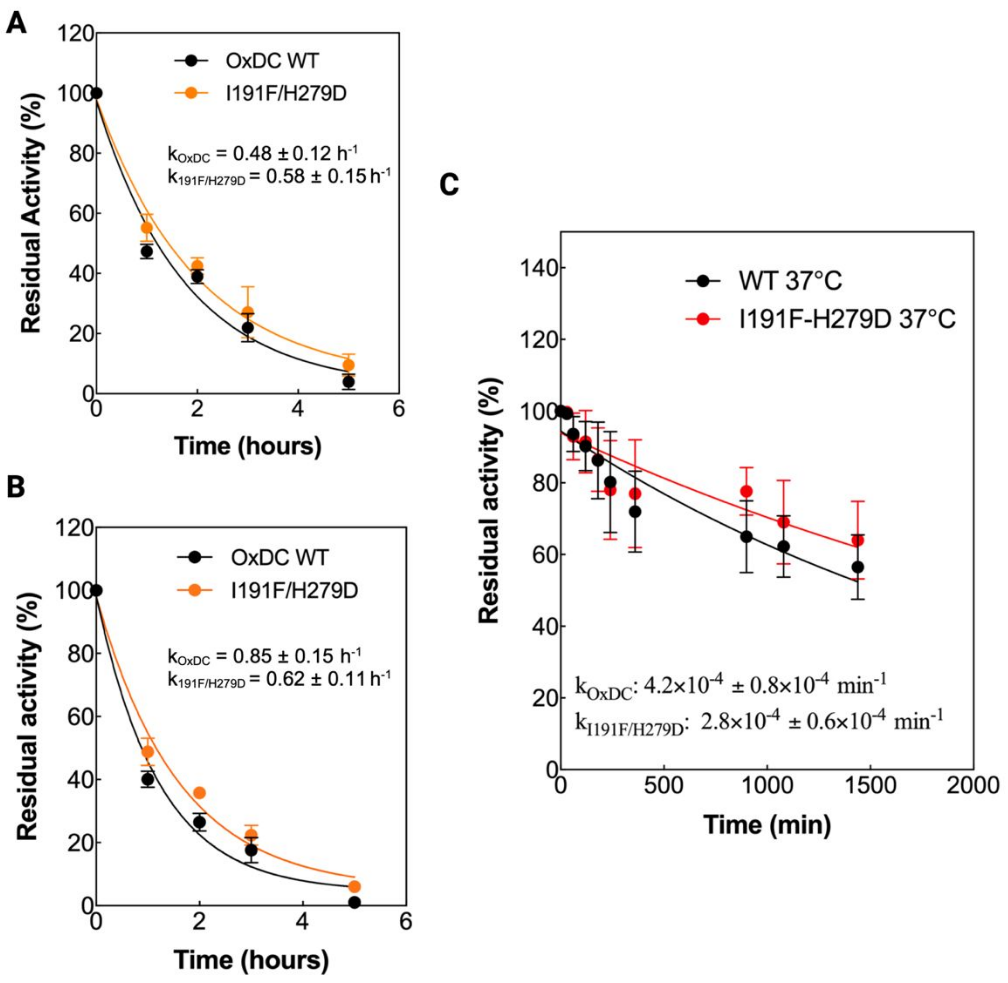

Figure S3. Residual activity plots in presence of two different proteases and under thermal stress at 37°C of OxDC WT and mutants I191F/H279D in PBS 1X pH 7.4. (A) and (B), Residual activity expressed as % of OxDC WT and double mutants I191F/H279D in presence of pancreatin (A) and α-chymotrypsin (B) used at a ratio 50:1 for 5 hours at 37°C. **(C)** Kinetic stability under thermal stress of OxDC WT and the mutant I191F/H279D at 37°C at different incubation times (total time 24 hours). Within the graphs are reported the rates values (k) obtained by fitting the experimental points using a single exponential decay function (see Material and Methods).

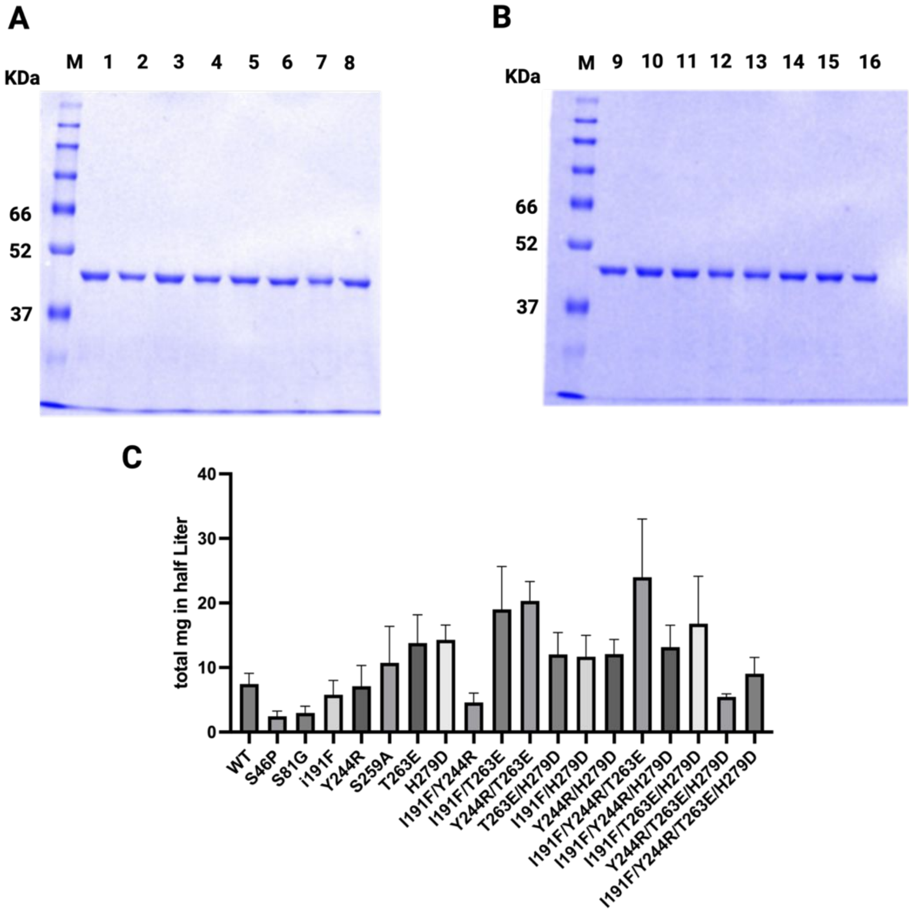

Figure S4. SDS–PAGE analysis of purified recombinant OxDC variants and yield of the purification. **Panel A and B:** SDS-PAGE gel of the purified proteins involved in the study. Lane M, protein marker, from 1 to 19 the purified proteins. In detail: 1: WT; 2: I191F; 3: Y244R; 4: T263E; 5: H279D; 6: I191F/Y244R; 7: I191F/T263E; 8: Y244R/T263E; 9: T263E/H279D; 10: I191F/H279D; 11: Y244R/H279D; 12: I191F/Y244R/T263E; 13: I191F/Y244R/H279D; 14: I191F/T263E/H279D; 15: Y244R/T263E/H279D; 16: I191F/Y244R/T263E/H279D. **Panel C:** yield expressed as total milligrams obtained from the purification of the proteins involved in this study.

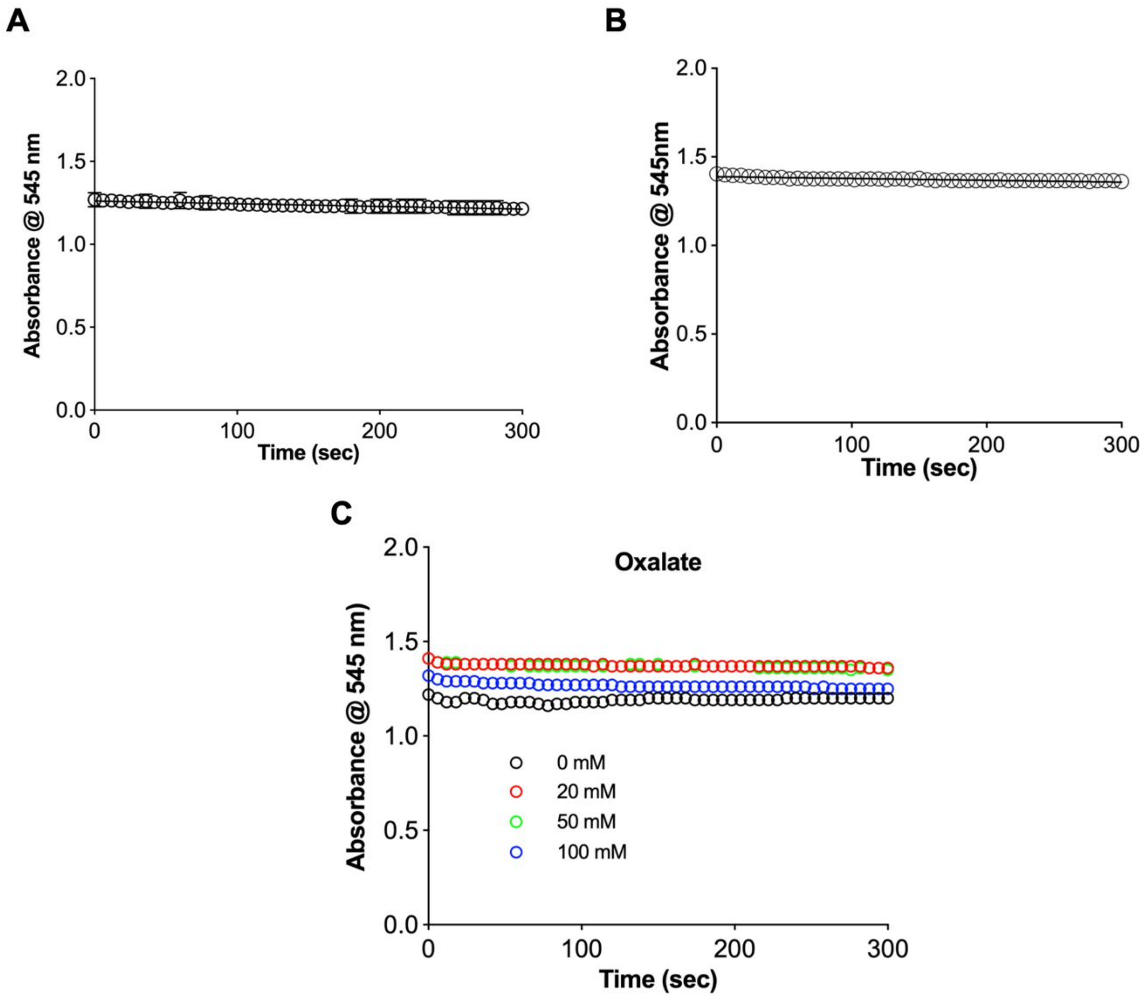

Figure S5. Set-up of the OxDC permanganate assay. A, Evaluation of the permanganate stability over time in KP 100 mM pH 8.0. **B,** Evaluation of the oxalate stability over time and its possible reaction with permanganate (100 mM) in presence of potassium permanganate 1 mM in KP 100 mM pH 8.0**. C,** Evaluation of the acetate interference (52 mM + 140 mM NaCl) over time in presence of potassium permanganate 1 mM in KP 100 mM pH 8.0.
